# Influence of Prosthetic Screw Designs with and Without Titanium Base on the Fracture Resistance of Implant-Supported Zirconia Frameworks

**DOI:** 10.3390/ma19214525

**Published:** 2026-10-27

**Authors:** Abdulrahman A. Maghrabi, Abdulaziz A. AlHelal, Abdulaziz S. Alqahtani, Syed Rashid Habib

**Affiliations:** Department of Prosthetic Dental Sciences, College of Dentistry, King Saud University, Riyadh 11545, Saudi Arabia; 444106197@student.ksu.edu.sa (A.A.M.); abalhelal@ksu.edu.sa (A.A.A.); absalqahtani@ksu.edu.sa (A.S.A.)

**Keywords:** aging, dental implant-abutment design, fracture strength, implant-supported prosthesis

## Abstract

This study aimed to evaluate the fracture resistance and patterns of implant-supported frameworks with and without a titanium base (Ti-base), using different prosthetic screw designs, and to determine how firing cycles followed by thermomechanical aging affect fracture resistance. Implant-supported zirconia frameworks were fabricated and allocated to three main groups: Ti-base with conventional screws, direct-connection with flat-seat screws, and direct-connection with conical screws. Each main group was further divided into two subgroups (*n* = 10). The control subgroup (C) did not undergo firing cycles or thermomechanical aging, whereas the experimental subgroup (E) underwent three consecutive firing cycles to simulate clinical zirconia veneering, followed by thermomechanical aging consisting of 5000 thermocycles and 1,250,000 chewing cycles. Fracture resistance differed significantly between screw designs in the control and experimental groups (*p* = 0.006 and *p* < 0.001, respectively). The combined effect of firing cycles and thermomechanical aging did not significantly affect fracture resistance in Ti-base and conical screw designs, whereas the flat-seat screw design showed a significant increase (*p* = 0.002). The failure pattern involved frequent deformation of prosthetic screws and multi-unit abutments (MUAs) in direct-connection designs. In contrast, the Ti-base design prevents such deformation, a benefit not present in direct-connection designs.

## 1. Introduction

Implant-supported fixed dental prostheses (ISFDPs) have become a highly successful option for restoring missing teeth [1,2]. Although metal-ceramic restorations were considered the gold standard because of their favorable mechanical properties and long-term clinical performance, computer-aided design and computer-aided manufacturing (CAD-CAM) have expanded the range of viable materials [3,4]. These technologies improve accuracy in data acquisition, design, and manufacturing, particularly for monolithic zirconia frameworks, which have demonstrated encouraging clinical performance [2,4,5]. Recent studies indicate that zirconia-based ISFDPs offer reliable long-term outcomes [3,4,5]. These materials are increasingly popular because of their enhanced biocompatibility, high fracture strength, toughness, and precision [6,7]. However, zirconia’s inherent brittleness and stiffness can transfer stress to the prosthesis-implant complex, leading to biological and technical complications [8,9,10].

In the posterior region, including molars and premolars, ISFDPs are required to withstand substantial masticatory forces to avoid mechanical failure [11,12,13]. Molars are subjected to higher occlusal forces, ranging from 300 Newtons (N) to 800 N, while anterior teeth are exposed to forces between 80 N and 200 N [12,14,15]. In individuals with parafunctional habits, occlusal loads may reach up to 1000 N [12,14].

Implant-supported monolithic zirconia frameworks have shown high long-term survival rates, as many clinical and retrospective studies support [16,17]. They often remain functional and clinically acceptable despite minor technical complications, including chipping of veneering ceramic, screw loosening, or non-catastrophic cracks [17]. Additional firing cycles during veneering and glazing, as well as exposure to thermomechanical cycling in simulated oral environments, can further influence the fracture resistance of zirconia frameworks [18,19,20,21,22,23]. Furthermore, failure modes and fracture patterns may differ for monolithic zirconia frameworks and other materials, such as titanium. Thus, these factors can affect restorative material selection and long-term survival [8,24].

In abutment-level prostheses, titanium interfaces known as Ti-base are frequently utilized with zirconia restorations [25]. These interfaces are cemented to the superstructure prosthesis, and screws secure them to the multi-unit abutment (MUA). Recent innovations in CAD-CAM technology and prosthetic screw design have enabled monolithic materials, such as zirconia, to be used directly on the MUA, eliminating the need for a Ti-base. This approach can increase framework bulk around the prosthetic screw head, reduce interfaces, and lower material costs. However, using conventional Ti-base screws directly on MUA is not recommended because zirconia and titanium exhibit distinct mechanical behaviors and require a minimum material thickness at the connection. To address this, modified prosthetic screw designs, such as the DESS flat-seat screw (DESS^®^, Barcelona, Spain) and the Vortex conical screw (Louisiana Implant Dental Lab, Los Angeles, CA, USA), have been developed to maintain adequate zirconia thickness at the screw head and MUA connection. Although recent innovations have emerged, the fracture resistance of these framework designs has not been thoroughly studied. Evidence on direct-connection ISFDPs remains limited, and framework fractures continue to pose a significant risk.

This in vitro study aimed to investigate the fracture resistance of various implant-supported framework interfaces, including Ti-base and zirconia direct-connections, using different prosthetic screw designs. Additionally, the study evaluated how additional firing cycles simulating the veneering process, followed by thermomechanical aging, affected fracture resistance. The study also assessed failure modes and fracture patterns at the framework, prosthetic screw, MUA, and implant levels for the different framework connections. The null hypotheses were: there would be no significant differences in fracture resistance and fracture pattern between direct-connection and Ti-base connection designs; geometric variations among the three prosthetic screw designs and the amount of milled zirconia at the screw head would not affect fracture resistance; and the combined effect of additional firing cycles and thermomechanical aging would not influence fracture resistance.

## 2. Materials and Methods

### 2.1. Study Design and Ethical Approval

This in vitro experimental study evaluated the fracture resistance and fracture patterns of zirconia implant-supported fixed dental prostheses with different framework–abutment interfaces and prosthetic screw designs, with and without veneering firing cycles, and before and after thermomechanical aging. The study protocol was reviewed and approved by the College of Dentistry Research Center (CDRC), approval number PR0195.

### 2.2. Sample Size

A power analysis was conducted using data from comparable in vitro studies. This calculation indicated that a minimum of 10 specimens per subgroup are required to achieve 80% statistical power at a 95% confidence level (α = 0.05), assuming an effect size of 0.5. Accordingly, each subgroup in this study consisted of 10 specimens.

### 2.3. Specimen Preparation

#### 2.3.1. Custom Mold Design and Fabrication

**Figure 1:**
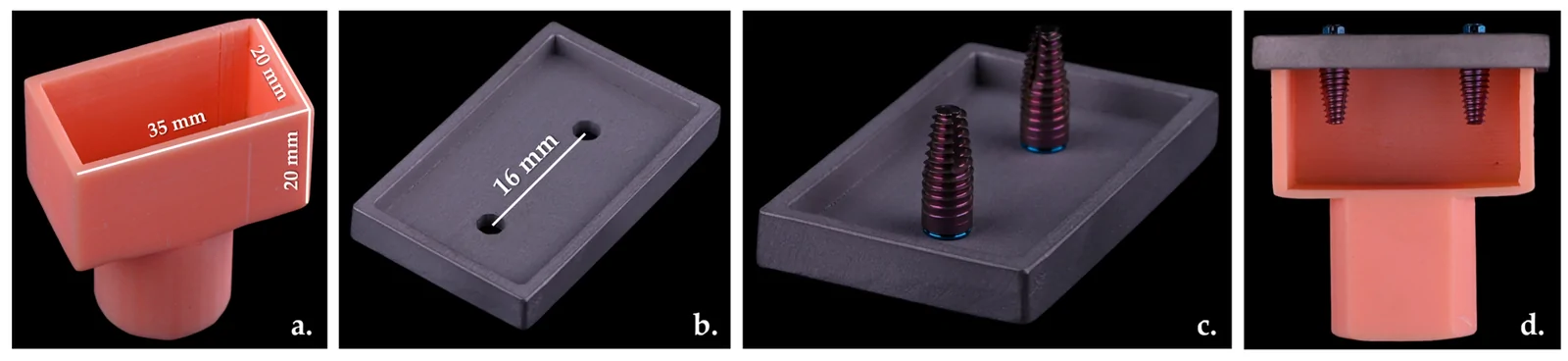
Custom-designed specimen mold fabrication components: (**a**) 3D-printed custom resin mold with specified dimensions; (**b**) 3D-printed custom metal cover with octagonal openings; (**c**) fully seated Loxim transfer pieces attached to implants; (**d**) cross-sectional view of the mounting assembly with embedded implants.

Table 1 lists all materials used throughout this study. To accommodate each three-unit implant-supported fixed dental prosthesis (two premolars and one molar), a total of sixty 3D-printed resin molds were fabricated, with each mold measuring 35 mm in length, 20 mm in width, and 20 mm in depth (Figure 1a). A cylindrical rod with a 20 mm diameter was incorporated into the base of each mold to allow secure seating in the chewing-simulation chamber holder. To ensure consistent vertical orientation of the implants across all specimens, the design also included a 3D-printed metal cover sized to match the mold, fitted with a flat vertical stop (Figure 1b,c). Horizontal implant positioning was standardized through a pair of centrally placed octagonal openings, set 16 mm apart, each shaped to receive the implant’s Loxim component (Loxim™ transfer piece, Straumann^®^, Basel, Switzerland) (Figure 1b,c). At a 2 mm metal thickness, this cover provided sufficient rigidity to securely support both the Loxim piece and the implant, keeping them stable throughout the embedding and mounting procedures (Figure 1c).

#### 2.3.2. Implants Mounting Procedure

Implants (Ø 4.1 mm × 10 mm, Bone Level Tapered, Regular CrossFit, Straumann^®^, Basel, Switzerland) were attached to the Loxim and the cover mold (Figure 1c). Following the manufacturer’s mixing protocol, clear acrylic resin (Major Ortho, Torino, Italy) was prepared and poured into the resin mold. With the implants still attached, the metal cover was seated onto the mold to ensure the implants were fully embedded in the acrylic resin (Figure 1d). The resin was allowed to cure for at least 24 h to ensure complete polymerization. The metal cover was subsequently detached once curing was complete, and the mold was cleaned of any leftover resin. This process was performed a total of sixty times to obtain the complete sample set.

### 2.4. CAD-CAM Framework Fabrication

#### 2.4.1. Framework Design

**Figure 2:**
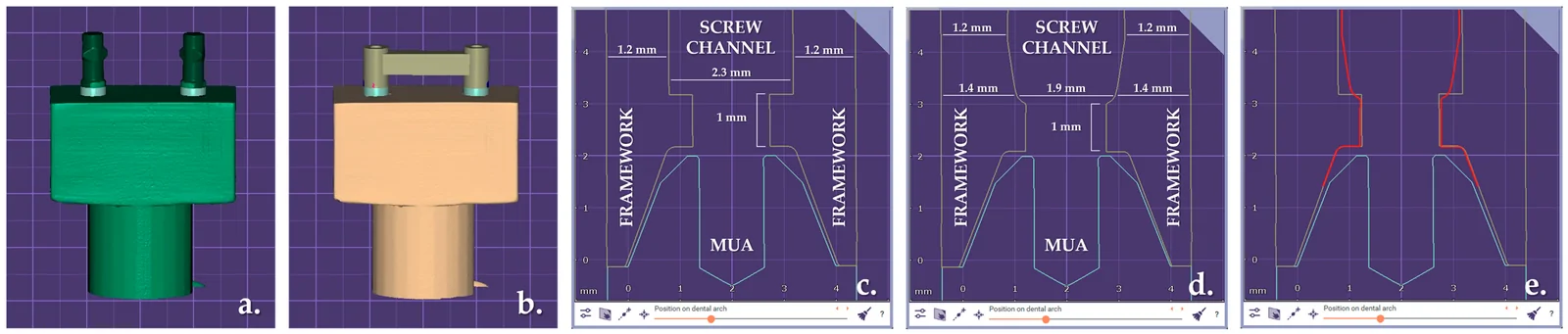
Specimen scanning and framework design: (**a**) scanned scan bodies and image superimposition in software to create a digital workflow model; (**b**) designed frameworks on the digital workflow model; (**c**) cross-sectional view of flat-seat screw design showing the thickness of zirconia material underlying the screw head; (**d**) cross-sectional view of conical screw design showing the thickness of zirconia material underlying the screw head; (**e**) cross-sectional view of superimposed two direct-connection designs showing the thickness difference of zirconia material underlying the screw head.

A straight multi-unit abutment (Regular CrossFit, Ø 4.6 mm, gingival height 1.5 mm; Straumann^®^, Basel, Switzerland) was selected for each implant and torqued into place using a digital torque gauge (Mark-10 MTT03; Mark-10, New York, NY, USA). To allocate the three-dimensional position of each abutment within the virtual model, multi-unit scan bodies (DESS^®^, Barcelona, Spain) were seated over the two MUAs and scanned using a Ceramill Map 600+ scanner (Amann Girrbach, Koblach, Austria). The resulting standard tessellation language (STL) files were imported into exocad DentalCAD v3.2 software (exocad GmbH, Darmstadt, Germany). Superimposition of the scanned images verified scan-body positioning and orientation, enabling accurate registration of each abutment’s 3D coordinates within the digital model (Figure 2a). Based on these coordinates, frameworks were designed as a 3-unit prosthesis with a connecting bar measuring 4 mm in both thickness and height (Figure 2b). Zirconia marginal thickness was standardized among all groups at 0.4 mm; a uniform framework thickness of 1 mm at the screw-head junction was adopted among all direct-connection screw design groups to maintain consistency in zirconia thickness. This value was dictated by the digital library for the flat-seat screw, which accommodates only a single, fixed thickness of 1 mm. In contrast, the conical screw design library permits a variable thickness ranging from 0.8 to 1.4 mm (Figure 2c,d,e).

#### 2.4.2. Framework Fabrication

**Figure 3:**
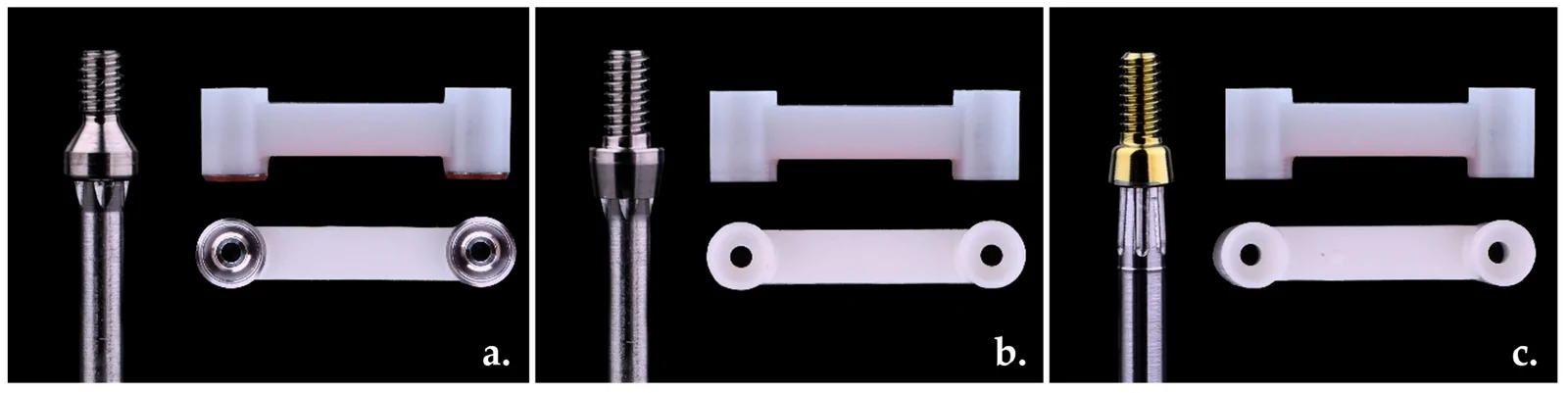
Screw designs and framework views: (**a**) Ti-base group with conventional screw; (**b**) Direct-connection group with flat-seat screw design; (**c**) Direct-connection group with conical screw design.

Zirconia frameworks were fabricated from 3 mol% yttria-stabilized zirconia blanks (Cercon^®^ base, Dentsply-Sirona, Hanau, Germany) using a 5-axis milling unit (Ceramill Mikro 5X, Amann Girrbach, Koblach, Austria), then sintered for 6 h at 1350 °C to achieve full strength. Frameworks in subgroup E underwent three sequential firing cycles to replicate the clinical veneering workflow: veneering firing at 780 °C, correction or adjustment firing at 770 °C, and final glaze firing at 750 °C. These cycles were conducted in a Programat P710 G2 furnace (Ivoclar Vivadent, Schaan, Liechtenstein) according to the manufacturer’s protocol for Celtra Ceram (Dentsply Sirona, Hanau, Germany). In the Ti-base group, titanium-base abutments (Ø 4.6 × 4 mm, Variobase^®^ for Bridge/Bar Cylindrical Coping; Straumann^®^, Basel, Switzerland) were bonded to the zirconia frameworks following the established bonding protocol [26,27]. The outer surface of the Ti-base and the internal surface of the zirconia framework were sandblasted with 50 µm Al_2_O_3_ (Corundum; Kulzer GmbH, Hanau, Germany) at 2 bar pressure from a distance of 10 mm for 10 s. Specimens were cleaned with 96% ethanol for 1 min, then air-dried. Clearfil ceramic primer plus (Kuraray Dental, Tokyo, Japan) was applied to the air-abraded zirconia and Ti-base surfaces. The zirconia framework was bonded to the corresponding Ti-base using Calibra Universal self-adhesive resin cement (Dentsply Sirona, Milford, DE, USA). Excess cement was removed from the interface with a dry microbrush. Oxyguard II (Kuraray Dental, Tokyo, Japan) was applied to the interface. Resin cement was then polymerized on each of the four sides for 20 s using a Demi Plus visible-light polymerization device (Kerr Corp., Brea, CA, USA). In direct-connection groups, frameworks were seated directly onto the multi-unit abutments with their corresponding screws (Figure 3a,b,c). Laboratory screws were used throughout specimen fabrication.

#### 2.4.3. Prosthetic Screw Groups Allocation

The specimens were allocated into three groups: zirconia frameworks with Ti-base connection using a conventional screw (Figure 3a), zirconia frameworks with direct-connection using a flat-seat screw head design (DESS^®^, Barcelona, Spain) (Figure 3b), and zirconia frameworks with direct-connection using a conical screw head design (Louisiana Implant Dental Lab, Los Angeles, CA, USA) (Figure 3c).

**Figure 4:**
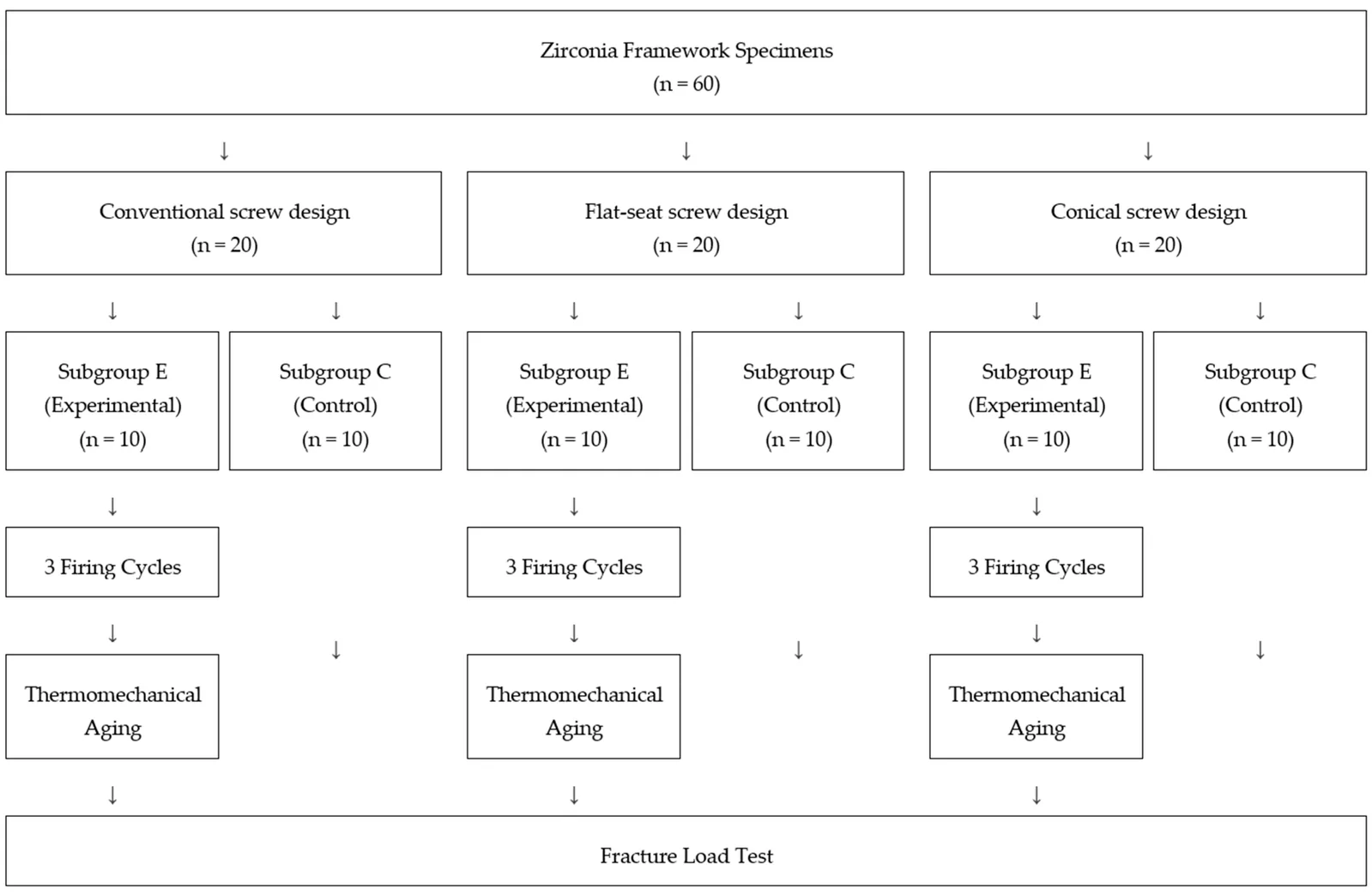
Schematic flowchart illustrating the distribution of prosthetic screw design groups and outlining the study workflow.

Each group consisted of two subgroups: an experimental group that underwent three firing cycles followed by thermomechanical aging (subgroup E), and a control group that was not exposed to firing cycles or thermomechanical aging (subgroup C), as shown in Figure 4. Specimens of each group were randomly allocated to the subgroups.

### 2.5. Framework Seating, Fit Verification, and Torquing

The MUA of each specimen was torqued to 35 Ncm per manufacturer specifications using a digital torque gauge (Mark-10 MTT03; Mark-10, New York, NY, USA), with a confirmation torque of 35 Ncm applied after 10 min. Frameworks of each specimen were seated, and the passive fit was assessed using a 1-screw Sheffield test and imaged with a NeoScan N70 μCT scanner (Neoscan BV, Mechelen, Belgium) to verify passive framework seating before further evaluation. Then, new prosthetic screws were introduced and torqued to 15 Ncm per manufacturer specifications using a digital torque gauge (Mark-10 MTT03; Mark-10, New York, NY, USA). A confirmation torque of 15 Ncm was applied after 10 min.

### 2.6. Thermomechanical Aging Process

**Figure 5:**
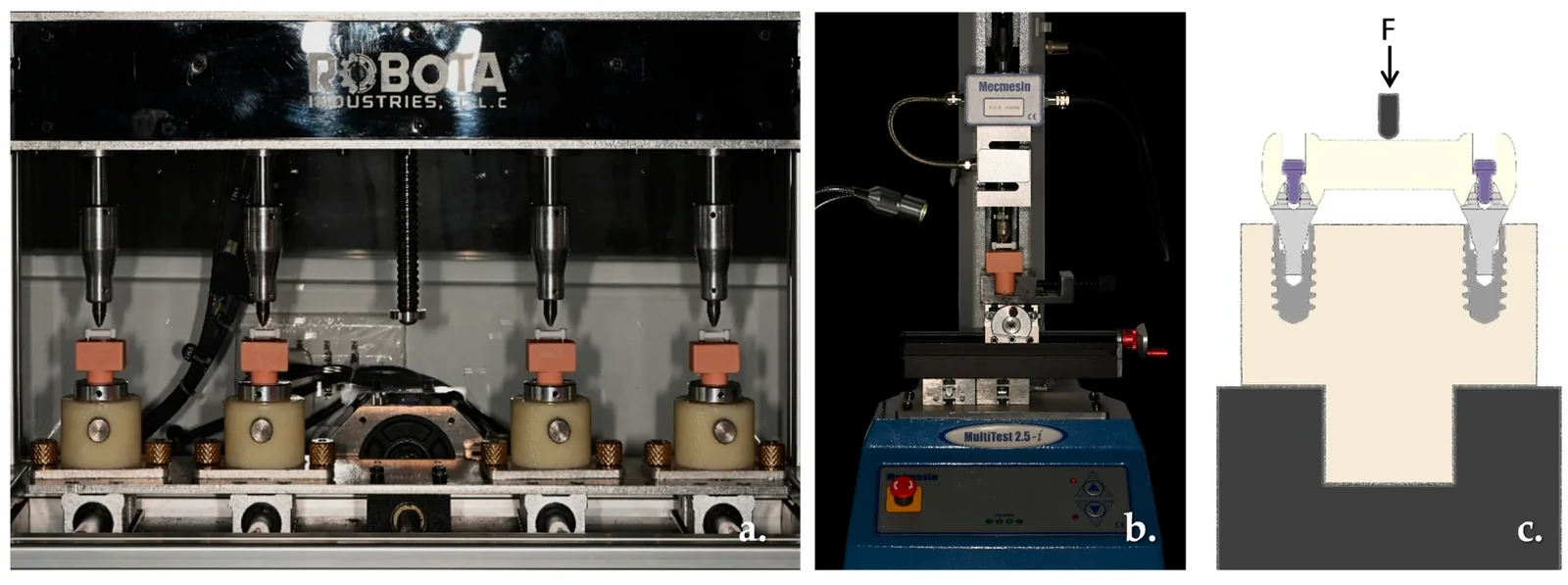
Experimental procedures: (**a**) Cyclic loading performed with a dual-axis chewing simulator. (**b**) Static load fracture performed with a universal testing machine. (**c**) Illustration of the mechanical testing assembly shows that the indenter forces act vertically towards the center of the framework, and the sample is self-centered in the machine holder through the extension.

After fabrication, specimens were subjected to thermocycling in a THE-1100 thermocycler (SD Mechatronik, Feldkirchen-Westerham, Germany) for 5000 cycles between 5 °C and 55 °C, with a 30 s dwell at each temperature and a 5 s transfer interval. Mechanical loading followed, using a four-station, dual-axis chewing simulator (Robota, AD-TECH Technology Co., Leinfelden-Echterdingen, Germany) for 1,250,000 cycles at 200 N (Figure 5a). The simulator features upper and lower crossbeams connected by two motor shafts: one controls the vertical movement of the upper crossbeam, which holds four antagonists, and the other controls the horizontal movement of the lower crossbeam, which holds four specimen chambers. Adjustable disk weights on guide rails allow the upper crossbeam to move freely vertically. Antagonist contact was achieved using a corrosion-resistant stainless steel (V4A) indenter with an 8 mm rounded tip. Loading parameters included a cycle frequency of 1.6 Hz, 2 mm vertical displacement, and 1 mm horizontal displacement. The load was applied vertically to the center of each framework along the implant’s long axis, with the specimen mold seated centrally in each crossbeam chamber (Figure 5c). Every 250,000 cycles, specimens were inspected for damage, such as cracks, fractures, or screw loosening, using a digital camera stereomicroscope (RaySmart Technology Co., Ltd., Shenzhen, China) at 20× magnification and a manual tactile motion check.

### 2.7. Fracture Load Test

After thermomechanical aging, framework specimens, prosthetic screws, and MUAs were inspected under a digital camera stereomicroscope at 20× magnification for cracks, thread delamination, or deformation. As no failures were observed, the same MUAs and prosthetic screws were retorqued to the corresponding torque value. All specimens that were exposed to thermomechanical aging, along with the remaining unexposed subgroup C, were tested for ultimate fracture load. This was done by applying a compressive vertical load along the implant axis until failure, using a MultiTest 2.5-i universal testing machine (Mecmesin Ltd., Slinfold, West Sussex, UK). A stainless-steel loading device with a 5 mm round tip was positioned at the midpoint of the zirconia framework. To ensure consistency, a custom positioning device was calibrated for each specimen holder to maintain an identical configuration prior to testing (Figure 5c). The testing load was a traditional load-to-failure test, using a static load that increased incrementally (1 mm/min in the vertical direction toward the frameworks) until fracture occurred (Figure 5b). The maximum load-to-fracture values were recorded in N using an Emperor™ Force software program (Mecmesin Ltd., Slinfold, West Sussex, UK) that captured the peak fracture loads for each specimen. Failure was defined as a sharp decrease in the force applied to the framework, as measured by the testing machine.

Post-test examination of all samples was conducted using a digital camera stereomicroscope (RaySmart Technology Co., Ltd., Shenzhen, China) at ×20 magnification to identify any catastrophic failures. Failure patterns were defined as follows: framework fracture at margin; prosthetic screw or MUA fracture; prosthetic screw or MUA deformation; framework separation from the substructure (with deformation); framework separation from the substructure (without deformation); implant fracture; and implant deformation.

### 2.8. Statistical Analysis

Data were collected and analyzed using IBM SPSS Statistics v26. Normality was assessed with the Shapiro–Wilk test, confirming a normal distribution. Results were reported as mean and standard deviation. Independent-samples *t*-tests evaluated differences between groups before and after thermomechanical aging. One-way ANOVA assessed differences among groups within the same time interval, followed by Tukey’s post hoc test to determine significance.

## 3. Results

Zirconia frameworks in both control (C) and experimental (E) groups exhibited significantly different mean fracture load values among the three prosthetic screw designs (*p* = 0.006 and *p* < 0.001, respectively), as shown in Table 2. In the control groups, the conical screw design recorded the highest mean fracture load (2070.87 ± 182.28 N), followed by the Ti-base screw design (1845.86 ± 267.68 N), and the flat-seat screw design exhibited the lowest mean value (1748.97 ± 158.61 N). Following firing cycles and thermomechanical cycling in the experimental groups, the conical screw design again yielded the highest mean fracture load (2121.24 ± 165.59 N), with the flat-seat screw design recording a substantially increased mean (1957.75 ± 78.08 N) and the Ti-base screw design demonstrating the lowest mean value (1740.37 ± 60.37 N).

Table 3 compares control and experimental groups within each screw design. For the Ti-base screw design, fracture load did not differ significantly between control and experimental conditions (*p* = 0.252). Similarly, the conical screw design showed no significant change after firing and thermomechanical cycling (*p* = 0.526). In contrast, the flat-seat screw design showed a statistically significant increase in fracture load after firing and thermomechanical cycling (*p* = 0.002), with experimental specimens exhibiting higher fracture resistance than their control counterparts.

Post hoc comparisons identified the specific pairwise differences that drove the significant ANOVA results, as shown in Table 4. In the control condition, the only statistically significant difference was between the flat-seat screw and conical screw designs (MD = −321.90 N; *p* = 0.005), with the conical screw design showing a significantly higher fracture load. In the control condition, the difference between the Ti-base screw and flat-seat screw designs (*p* = 0.558), as well as between the Ti-base screw and conical screw designs (*p* = 0.057), did not reach statistical significance. In the experimental condition, the Ti-base screw design showed a significantly lower fracture load than both the flat-seat screw design (MD = −217.38 N; *p* < 0.001) and the conical screw design (MD = −380.87 N; *p* < 0.001). The flat-seat screw design also showed a significantly lower fracture load than the conical screw design (MD = −163.49 N; *p* = 0.008).

**Figure 6:**
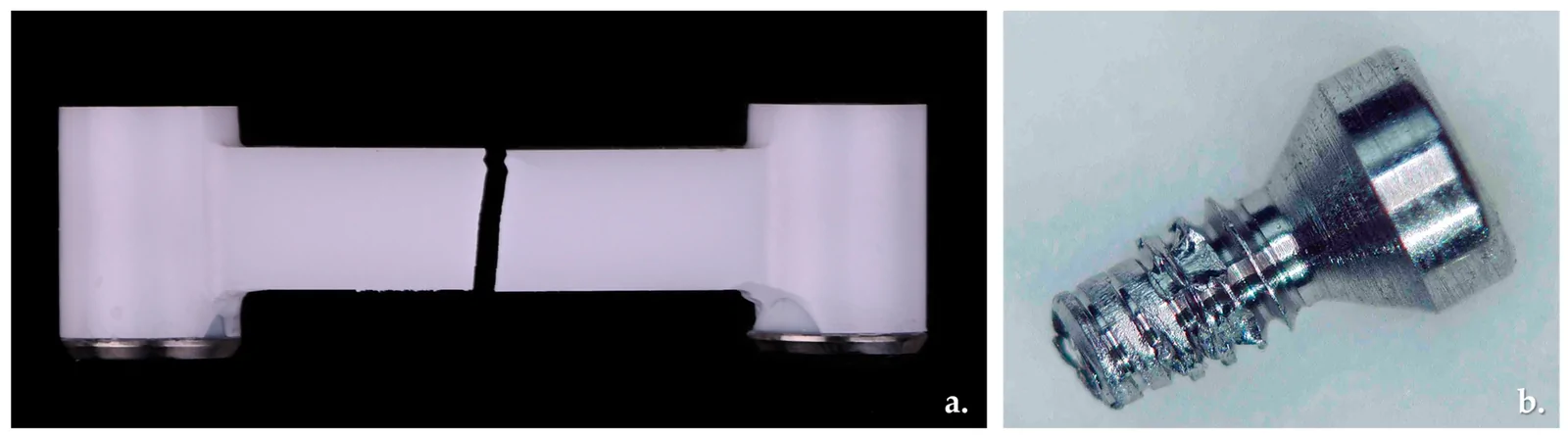
Failure patterns of the Ti-base screw design: (**a**) Framework fracture at the pontic and margin; (**b**) prosthetic screw deformation.

**Figure 7:**
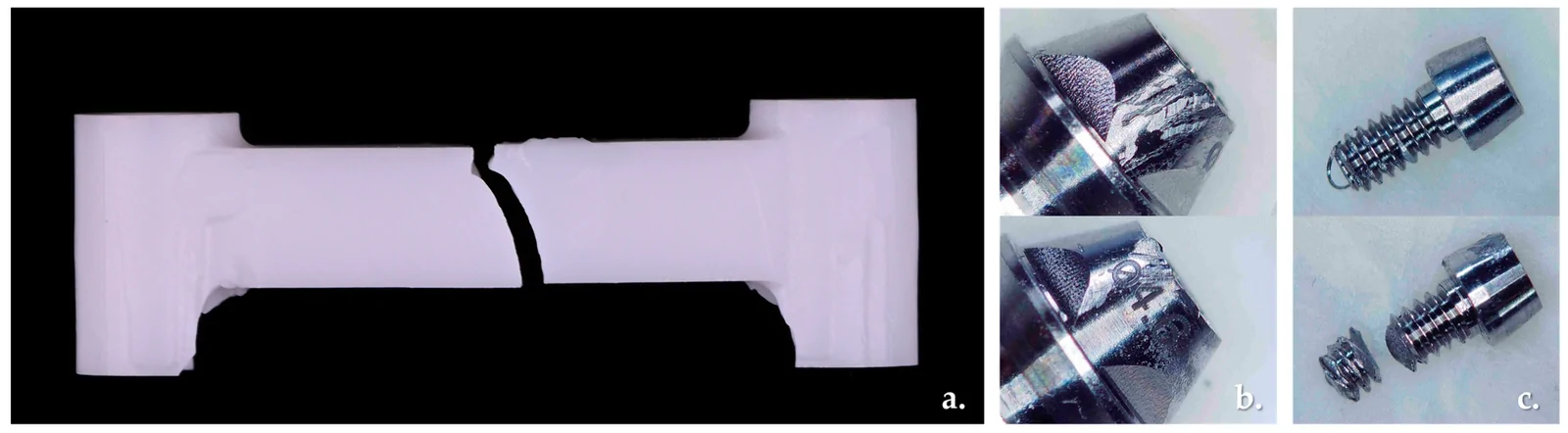
Failure patterns of the flat-seat screw design: (**a**) Framework fracture at the pontic and margin; (**b**) MUA deformation; (**c**) prosthetic screw deformation and fracture.

**Figure 8:**
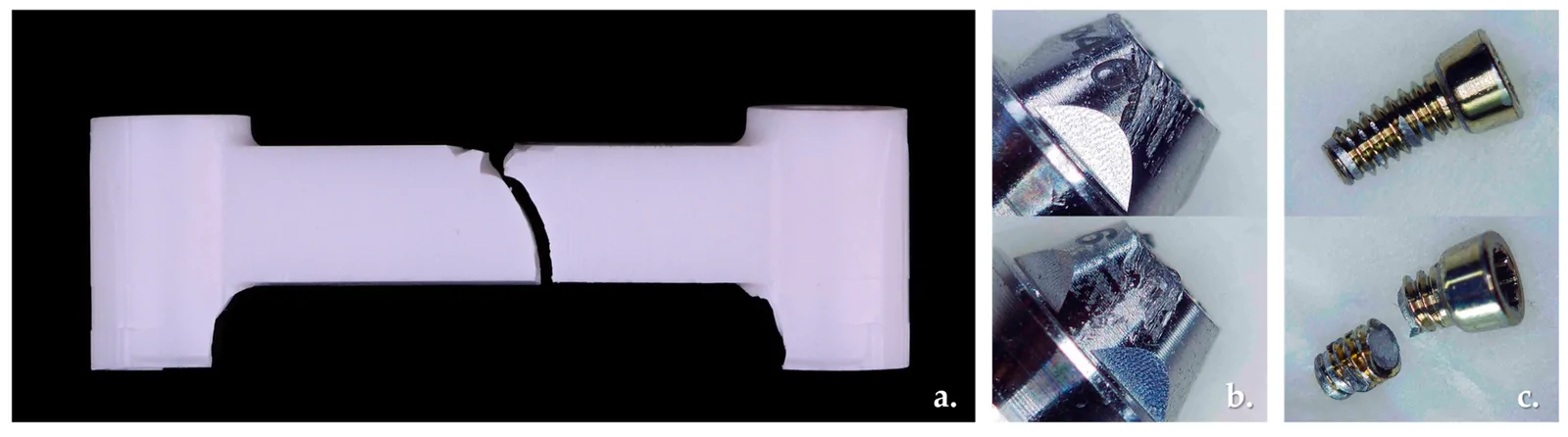
Failure patterns of the conical screw design: (**a**) Framework fracture at the pontic and margin; (**b**) MUA deformation; (**c**) prosthetic screw deformation and fracture.

Table 5 and Figure 6, Figure 7 and Figure 8 present the failure patterns observed in the zirconia frameworks of the control and experimental groups among the three screw designs. Prosthetic screw deformation was frequent in most flat-seat and conical designs, but occurred in only one screw in the Ti-base design. However, the extent of screw deformation ranged from slight delamination of the screw thread to screw threads bending. Prosthetic screw fracture occurred infrequently among all groups, with the highest incidence in direct-connection designs. MUA deformation affected all specimens in the flat-seat and conical designs but was absent in the Ti-base design.

## 4. Discussion

The results of this study support rejecting the first two null hypotheses: fracture load differed significantly between direct-connection design and the Ti-base design, and fracture load differed significantly between direct-connection frameworks based on screw head design and the amount of milled zirconia at the screw head seat. The third null hypothesis was partially rejected. Firing cycles followed by thermomechanical aging significantly influenced fracture load only in the flat-seat screw design.

The results indicate that the control conical direct-connection framework design had higher fracture resistance than the control Ti-base design, whereas in the experimental groups, both direct-connection framework designs exceeded the Ti-base design, with the effect being most observed in the conical screw design. Although this increase was not significant in the control groups, it was significant in the experimental groups. This outcome can be explained by the greater bulk thickness of the direct-connection frameworks at the screw head seat, resulting from the absence of the Ti-base, thereby enhancing occlusal load resistance. In this study, fracture resistance for the various frameworks ranged from 1748.97 N to 2121.24 N. These values exceed physiologic maximum occlusal forces in the posterior region, which typically range from 300 to 800 N and can reach 1000 N in patients with bruxism [12,14,15]. However, these observations have limited applicability to clinical situations because the study employed constant occlusal forces regarding magnitude, duration, application point, and angulation. Furthermore, the study did not account for variables inherent to the oral environment, such as saliva, the effects of biting hard objects, temperature fluctuations, bone quality, implant positioning and angulation, framework misfit, and screw preload loss.

The control direct-connection flat-seat screw design showed significantly lower fracture resistance than the control direct-connection conical design. This reduction likely results from the prosthetic screw head design and the extent of milling required at the screw channel and screw head seat. Specifically, the flat, wide screw head base requires more zirconia milling at the seat and channel to accommodate the screw, as previously illustrated in Figure 2e. In contrast, the tapered conical design maintains greater zirconia thickness at both the screw head seat and channel. However, after firing cycles and thermomechanical aging simulating clinical conditions, flat-seat zirconia showed increased fracture resistance, exceeding that of the Ti-base design and the physiologic biting forces.

The results indicate that firing cycles followed by thermomechanical aging did not significantly decrease fracture resistance in the Ti-base screw design, nor did they significantly increase it in the conical screw design. However, the flat-seat screw design showed a significant increase. These findings suggest that additional firing cycles, which simulate zirconia veneering and staining procedures, did not significantly affect fracture resistance. Previous studies indicate that repeated zirconia firing increases the fracture resistance of veneered ceramics [20] and improves the framework’s mechanical properties, including phase transformation, hardness, and porosity [28,29,30]. These changes may hypothetically account for the increased load resistance observed in the experimental direct-connection designs. To confirm this explanation, fracture load should be assessed after firing cycles alone, without the influence of thermomechanical aging. Additionally, several studies have demonstrated that thermomechanical aging does not significantly affect fracture resistance of monolithic zirconia [30,31,32,33]. The observed decrease in fracture resistance in the experimental Ti-base screw design may hypothetically be attributed to reduced bond strength between the zirconia framework and the titanium base due to thermomechanical aging, since bond strength was not the scope of the study and was not assessed. However, previous studies show that reduced bond strength can adversely affect fracture resistance [34,35,36,37,38].

Multiple comparisons between the two direct-connection designs showed that the conical direct-connection design had significantly higher fracture resistance than the flat-seat designs in both the control and experimental conditions. As previously discussed, at a constant zirconia framework thickness, the conical screw design performs better because it preserves greater zirconia thickness at the screw head seat and channel than the flat-seat design. Therefore, when using flat-seat screw designs, it is recommended to increase the framework thickness at the margin and connector to compensate for the reduced thickness required by the screw head design.

Failure patterns varied between the Ti-base and direct-connection designs. In the Ti-base, failures were mainly limited to the frameworks, with only one incidence of prosthetic screw deformation. No MUA deformation or prosthetic screw fracture was observed. In contrast, the direct-connection designs showed more frequent MUA and prosthetic screw deformation. Some prosthetic screws in these designs also showed prosthetic screw fracture. This variation in failure patterns is attributed to the Ti-base absorbing forces applied to the zirconia framework before they reach the prosthetic screws and MUAs. In direct-connection designs, forces are transmitted directly from the zirconia framework to the underlying structures, including the prosthetic screws and MUAs. Titanium can withstand higher occlusal forces than zirconia due to zirconia’s brittle nature [24]. Therefore, the Ti-base prevents deformation of the prosthetic screw and MUA, a benefit not present in direct-connection designs. Furthermore, because the Ti-base screw design fractured at a lower mean load than direct-connection designs, this reduced load resistance could be considered a failure pattern for the Ti-base design.

This study has several limitations. First, the in vitro design limits clinical relevance, as it does not incorporate biological variables such as saliva flow, bone density, or remodeling. Second, the study performed only static load testing after thermomechanical aging with one loading position, whereas progressive dynamic fatigue testing would better simulate clinical conditions and may provide more accurate predictions of clinical outcomes. Third, the study used a single standard specimen dimension, although variations in pontic design and margin thickness could affect the ultimate fracture force. Additionally, the study did not assess the effect of ceramic firing alone on fracture resistance and fracture pattern. The study also did not provide fractographic evidence. Microscopic examination would help assess fracture, crack initiation, and localized deformation. Further in vitro, in vivo, and clinical studies with longer follow-up, larger sample sizes, and varied conditions are needed to evaluate the feasibility of modified prosthetic screws.

## 5. Conclusions

Based on the results of this in vitro study, the following conclusion could be drawn:

- Direct-connection implant-supported zirconia frameworks showed significantly greater fracture resistance than Ti-base frameworks after thermomechanical aging. The modified conical screw design offered the highest fracture resistance.
- The combined effect of firing cycles and thermomechanical aging did not significantly influence fracture resistance in the Ti-base and modified conical screw design frameworks; however, it significantly increased fracture resistance in the modified flat-seat screw design framework.
- Failure patterns varied by framework design. The Ti-base design shows no deformation in the prosthetic screw or MUA, whereas direct-connection designs transmit forces directly to the prosthetic screw and MUA, causing deformation.

## Figures and Tables

**Table 1:** Details of all materials utilized in the study.

Test Material	Brand	Manufacturer	Composition
3D-printed resin	SG 5.0 resin, Arum Dentistry	ARUM Dentistry Co., Ltd., Daejeon, Republic of Korea	Urethane dimethacrylate/UDMA, reactive monomers/TEGDMA-HEMA, photoinitiator, stabilizer, orange pigment.
3D-printed metal	Audental SLM CoCr Dental Powder	Audental Bio-Material Co., Ltd., Shanghai, China	Co ~61.5%, Cr ~28.1%, Mo ~5.0%, W ~5.3%
Implant Loxim	Loxim™ transfer piece, Straumann	Straumann^®^, Basel, Switzerland	-
Implant	Ø 4.1 mm × 10 mm, Bone Level, Regular CrossFit, Straumann	Straumann^®^, Basel, Switzerland	Titanium
Acrylic resin	Major.Ortho	Major Ortho, Torino, Italy	Powder: Polymethylmethacrylate, benzoyl peroxide initiator, colorants. Liquid: Methyl methacrylate monomer, tertiary amine/DMPT accelerator, hydroquinone stabilizer.
Multi-unit abutment	Ø 4.6 mm, Regular CrossFit, Gingival Height 1.5 mm	Straumann^®^, Basel, Switzerland	Titanium
Titanium Variobase	Ø 4.6 mm × 4 mm Variobase^®^ for Bridge Cylindrical	Straumann^®^, Basel, Switzerland	Titanium
Occlusal prosthetic screw	Occlusal screw, for coping, screw-retained abutment	Straumann^®^, Basel, Switzerland	Titanium Head diameter: 2.2 mm, head length: 1.5 mm, head base angle: 60°, thread length: 2.2 mm, thread type: M1.4, total screw length: 3.7 mm
Flat-seat prosthetic screw	DESS^®^ New Flat Seat Screw	DESS^®^, Barcelona, Spain	Titanium Head diameter: 2.3 mm, head length: 1.4 mm, head base angle: 0°, thread length: 2.7 mm, thread type: M1.4, total screw length: 4.1 mm
Conical prosthetic screw	Vortex™ Direct to MUA Screw	Louisiana Implant Dental Lab, Los Angeles, CA, USA	Titanium Head diameter: 2.1 mm, head length: 1.4 mm, head base angle: 14°, thread length: 2.8 mm, thread type: M1.4, total screw length: 4.2 mm
3Y Zirconia	Cercon^®^ base	Dentsply-Sirona, Hanau, Germany	ZrO_2_ ~92%, Y_2_O_3_ 5%, HfO_2_ 2%, Al_2_O_3_ and SiO_2_ < 1%
Airborne particle abrasive	Hera^®^ Corundum	Kulzer GmbH, Hanau, Germany	50 μm, Aluminum Oxide (Al_2_O_3_)
Primer	Clearfil™ ceramic primer plus	Kuraray, Tokyo, Japan	3-MPS, 10-MDP, Ethanol
Universal Self-Adhesive Resin Cement	Calibra^®^ Universal	Dentsply-Sirona, Milford, DE, USA	Base and catalyst: UDMA, TEGDMA, TMPTMA, HEMA, and Bis-EMA, with inorganic filler; catalyst also contains PENTA.

**Table 2:** Fracture load (N) of zirconia framework groups among prosthetic screw designs.

Groups		M ± SD	Min	Max	95% CI		*p*-Value *
					Lower	Upper	
C	Ti-base screw (*n* = 10)	1845.86 ± 267.68	1492.30	2385.40	1654.37	2037.34	0.006
	Flat-seat screw (*n* = 10)	1748.97 ± 158.61	1410.80	1926.40	1635.50	1862.34	
	Conical screw (*n* = 10)	2070.87 ± 182.28	1703.20	2336.00	1940.47	2201.26	
E	Ti-base screw (*n* = 10)	1740.37 ± 60.37	1637.80	1864.70	1697.17	1783.56	<0.001
	Flat-seat screw (*n* = 10)	1957.75 ± 78.08	1817.80	2108.30	1901.89	2013.60	
	Conical screw (*n* = 10)	2121.24 ± 165.59	1766.90	2351.60	2002.78	2239.69	

C, control group; E, experimental group; M ± SD, mean and standard deviation; Min, minimum; Max, maximum; 95% CI, 95% confidence interval of the mean. * *p*-value was significant at *p* < 0.05 for one-way ANOVA test.

**Table 3:** Comparison of fracture load (N) between control and experimental zirconia framework groups among prosthetic screw designs.

	C M ± SD (*n* = 10)	E M ± SD (*n* = 10)	95% CI		t	*p*-Value *
			Lower	Upper		
Ti-base screw (*n* = 10)	1845.86 ± 267.68	1740.37 ± 60.37	−88.08	299.06	1.216	0.252
Flat-seat screw (*n* = 10)	1748.97 ± 158.61	1957.75 ± 78.08	−326.23	−91.32	−3.734	0.002
Conical screw (*n* = 10)	2070.87 ± 182.28	2121.24 ± 165.59	−213.98	113.24	−0.647	0.526

C, control group; E, experimental group; M ± SD, mean and standard deviation; 95% CI, 95% confidence interval of the mean difference. * *p*-value was significant at *p* < 0.05 for Independent-samples *t*-test.

**Table 4:** Post hoc comparison of fracture load (N) of zirconia framework groups among prosthetic screw designs.

Groups			MD	Std. Error	*p*-Value *	95% CI	
						Lower	Upper
C	Ti-base screw	Flat-seat screw	96.8900	93.1088	0.558	−133.9658	327.7458
		Conical screw	−225.0100	93.1088	0.057	−455.8658	5.8458
	Flat-seat screw	Conical screw	−321.9000	93.1088	0.005	−552.7558	−91.0442
E	Ti-base screw	Flat-seat screw	−217.3800	49.7750	<0.001	−340.7932	−93.9668
		Conical screw	−380.8700	49.7750	<0.001	−504.2832	−257.4568
	Flat-seat screw	Conical screw	−163.4900	49.7750	0.008	−286.9032	−40.0768

* *p*-value was significant at *p* < 0.05.

**Table 5:** Failure patterns of zirconia framework groups among prosthetic screw designs.

Groups	Framework Fracture at Margin	Ti-Base Debonding	Prosthetic Screw Deformation	Prosthetic Screw Fracture	MUA Deformation
Ti-base screw (C)	7/10	0/10	1/10	0/10	0/10
Ti-base screw (E)	5/10	0/10	0/10	0/10	0/10
Flat-seat screw (C)	10/10	-	9/10	1/10	10/10
Flat-seat screw (E)	10/10	-	8/10	2/10	10/10
Conical screw (C)	9/10	-	8/10	1/10	10/10
Conical screw (E)	8/10	-	8/10	1/10	10/10

C, control group; E, experimental group.

## Data Availability

The data are available upon request by contacting the corresponding author through e-mail.

## Abbreviations

The following abbreviations are used in this manuscript:

Ti-base	Titanium base
C	Control group
E	Experimental group
MUAs	Multi-unit abutments
ISFDPs	Implant-supported fixed dental prostheses
CAD-CAM	Computer-aided design and computer-aided manufacturing
N	Newton
STL	Standard tessellation language
